# Inhibition of the mTOR Pathway Exerts Cardioprotective Effects Partly through Autophagy in CLP Rats

**DOI:** 10.1155/2018/4798209

**Published:** 2018-06-28

**Authors:** Wen Han, Hao Wang, Longxiang Su, Yun Long, Na Cui, Dawei Liu

**Affiliations:** Department of Critical Care Medicine, Peking Union Medical College Hospital, Peking Union Medical College and Chinese Academy of Medical Science, Beijing 100730, China

## Abstract

**Background:**

Sepsis-induced myocardial dysfunction is a severe clinical problem. Recent studies have indicated that autophagy and myocardial energy depletion play a major role in myocardial dysfunction during sepsis, a mechanistic target of rapamycin (mTOR) as a master sensor of energy status and autophagy mediator; however, there are little data describing its role during sepsis in the heart.

**Methods:**

Cecal ligation and puncture (CLP) or sham operation (SHAM) was performed in rats. After treatment, pathological changes were determined by H&E staining, cardiac functions by echocardiography, the distribution of microtubule-associated protein light chain 3 (LC-3) type II and hypoxia-inducible factor 1*α* (HIF-1a) by immunohistochemical staining, and autophagic vacuoles by transmission electron microscopy. Moreover, the mTOR signaling pathway and LC3II, p62, and HIF-1a expression were measured by western blotting.

**Results:**

Rapamycin alleviated the pathological damage of myocardial tissue, attenuated cardiac dysfunction (left ventricular ejection fraction (LVEF), *p* < 0.05; fractional shortening (FS), *p* < 0.05), and reduced HIF-1a expression (*p* < 0.05). Expectedly, rapamycin decreased the activity of the mTOR pathway in both sham-operated rats (*p* < 0.0001) and CLP rats (*p* < 0.01). Interestingly, we also found inhibition of the mTOR pathway in CLP rats compared with sham-operated rats; phosphorylation of both mTOR (*p* < 0.001) and pS6K1 (*p* < 0.01) was significantly suppressed following CLP challenge. Furthermore, autophagic processes were elevated by CLP; the ratio of LC3II/LC3I (*p* < 0.05) was increased while p62 expression (*p* < 0.001) was decreased significantly; there were also more autophagic vacuoles in CLP rats; and rapamycin could further elevate the autophagic processes compared with CLP rats (LC3II/LC3I, *p* < 0.05; P62, *p* < 0.05).

**Conclusion:**

Inhibition of the mTOR pathway has cardioprotective effects on myocardial dysfunction during sepsis induced by CLP, which is partly mediated through autophagy.

## 1. Introduction

Sepsis as a systemic response to infection is a leading cause of morbidity and mortality worldwide. Despite advances in critical care treatment and increased understanding of the sepsis pathophysiology, the mortality rate of affected patients remains high (40%–60%), even in developed countries [1]. High mortality in septic patients is associated with cardiac dysfunction. When people suffer from severe heart failure, their mortality rate increases by 50% [2]. To develop more effective therapies for septic myocardial dysfunction, it is necessary to explore the mechanisms of cardiac dysfunction induced by sepsis.

Autophagy refers to any cellular degradative pathway that involves delivery of cytoplasmic cargo to the lysosome [3]. LC3-II and p62 are two major proteins in autophagy. LC3II functions at an early stage of phagophore expansion, while p62/SQSMT1 has an adaptor function to recognize ubiquitinated proteins that need to be removed from the cytoplasm during autophagy; its amount is generally considered to inversely correlate with autophagic activity [4–6]. In the basal state, autophagy is an important process in the heart, and a defect in this process can be detrimental [7]. It also plays an important role in the modulation of ischemia-reperfusion (I/R) injury. Evidence suggests that autophagy protects against I/R injury, and impaired autophagy contributes to higher morbidity and mortality [8]. Recent studies have found that autophagy also plays an important role in septic myocardial depression. Accumulating evidence has shown that autophagic activity in cardiomyocytes changes during sepsis, but the results are unclear [9, 10].

Mammalian target of rapamycin (mTOR) is a master sensor of energy status and promotes autophagy when there is energy depletion [11]. Myocardial energy depletion plays a major role in myocardial dysfunction during sepsis [12, 13]. However, to the best of our knowledge, there is little data describing the role of the mTOR pathway during sepsis in the heart [10, 14]. Furthermore, the relationship between autophagy and the mTOR pathway in cardiac dysfunction caused by sepsis is still unknown, which was investigated in this study.

## 2. Methods

### 2.1. Animal Model

Healthy male Wistar rats, weighing 250 ± 10 g, were obtained from the Animal Facility Center, PUMCH. All animals were housed in a pathogen-free facility and treated according to protocols approved by the Institutional Animal Care and Use Committee of PUMCH. Rats were randomly divided into SHAM, SHAM + rapamycin (RAPA), cecal ligation and puncture (CLP), and CLP + RAPA groups (six mice per group). CLP was performed as previously described to establish a mid-grade sepsis model [15]. In brief, under chloral hydrate anesthesia, the cecum was exposed by a 3 cm midline laparotomy. The mesentery of the cecum was carefully dissected, and the cecum was ligated at half the distance between the distal pole and base of the cecum. Two cecal punctures were made with a 22 G needle, and a droplet of feces was forced out from both the mesenteric and antimesenteric penetration holes to ensure patency of the punctures. Sham-operated rats were subjected to the same laparotomy without CLP. After surgery, all animals immediately received a subcutaneous injection of sterile saline (0.9% NaCl, 5 ml per 100 g body weight) for resuscitation. Eighteen hours after surgery, the animals were killed, and the hearts were removed for further evaluation. In treatment groups, rapamycin (10 mg/kg BW) was administered intraperitoneally for the 7 consecutive days before CLP operation. In the vehicle-treated group, mice received the same volume of vehicle (10% DMSO, 4 ml/kg BW) intraperitoneally. The dose of rapamycin was chosen based on a previous study [9].

### 2.2. Hematoxylin and Eosin (H&E) Staining

The left ventricle hearts of rats were transversely cut at a 2 mm thickness, immediately fixed in 4% paraformaldehyde, and embedded in paraffin. Sections of 3 *μ*m in thickness were affixed to slides, deparaffinized, and stained with H&E to evaluate morphological changes of the heart.

### 2.3. Transmission Electron Microscopy Analysis

Transmission electron microscopy was performed as described previously [16]. The heart was perfused with 0.5% glutaric dialdehyde in 0.1 M cacodylate buffer (perfusion fluid) at 3 ml/min for 5 min to fix the muscle in situ. Then, the freshly isolated cardiac tissue from mice was cut into small pieces and immediately fixed by immersion in perfusion fluid for 3 h. After washing with 0.1 M cacodylate buffer and 15% sucrose buffer, the samples were cut into thin sections (90 nm) that were viewed at 120 kV with a H7650 transmission electron microscope (HITACHI, Tokyo, Japan). Micrographs were obtained using a Philips CM12 (10–15 per sample) by random sampling.

### 2.4. Western Blot Analysis

The left ventricle tissues were homogenized in lysis buffer. Tissue lysates were centrifuged at 17,000*g* for 10 min. An aliquot of the supernatant was used to determine the protein concentration. Protein samples were mixed with 4× lithium dodecyl sulfate sample buffer, electrophoresed on SDS-polyacrylamide gels, and then transferred electrophoretically onto nitrocellulose. The membranes were immunoblotted with antibodies against LC3B (ab48394, Abcam, 1 : 1000 dilution), p62 (#9234, Cell Signaling Technology, 1 : 1000 dilution), phospho-mTOR (Ser2448) (#5536, Cell Signaling Technology, 1 : 8500 dilution), phospho-p70S6 kinase (Thr389) (#9234, Cell Signaling Technology, 1 : 500 dilution), HIF-1a (ab2185, Abcam, 1 : 1000 dilution), and actin. A horseradish peroxidase-conjugated goat anti-rabbit secondary antibody was used. After the final wash, the membranes were developed using enhanced chemiluminescence (Amersham, Piscataway, NJ) and autoradiographed. Actin was used as a loading control.

### 2.5. Immunohistochemistry

Formalin-fixed, paraffin-embedded sections of rat hearts were subjected to immunohistochemical analysis as described previously [17]. Briefly, 3 *μ*m thick tissue sections were deparaffinized and rehydrated, followed by antigen retrieval in a microwave, according to the standard procedures. The sections were sequentially blocked with avidin and biotin and then incubated at 4°C overnight with a 1/1000-diluted anti-LC3 antibody (ab48394, Abcam) and 1/1000-diluted anti-HIF-1a antibody (ab2185, Abcam). Following repeated washes, the sections were incubated at 25°C for 30 min with biotinylated IgG. Diaminobenzidine was used as a development substrate. The sections were dehydrated and mounted with DePex.

### 2.6. Echocardiography Examination

Rats were anaesthetized intraperitoneally with pentobarbital (70–80 mg/kg) at 18 h after CLP and situated in the supine position on a warming pad. An ultrasonic machine (M-Turbo Sonosite, USA) equipped with a 15 MHz transducer was used for noninvasive transthoracic echocardiography. The left ventricular end-diastolic and end-systolic dimensions were measured. The left ventricular ejection fraction (LVEF) and fractional shortening (FS) were also calculated from M-mode echocardiograms. Data from three consecutive selected cardiac cycles were analyzed and averaged.

### 2.7. Statistical Analysis

Data were analyzed by SPSS 18.0 software (SPSS Inc., IBM Corp., Armonk, NY, USA). All data for continuous variables in this study had normal distributions and are shown as the mean ± standard deviation. Differences were assessed using analysis of variance followed by the least significant difference (LSD). A *p* value of less than 0.05 was considered as statistically significant.

## 3. Results

### 3.1. Pathological Changes in the Myocardial Tissue of Model Rats

**Figure 1:**
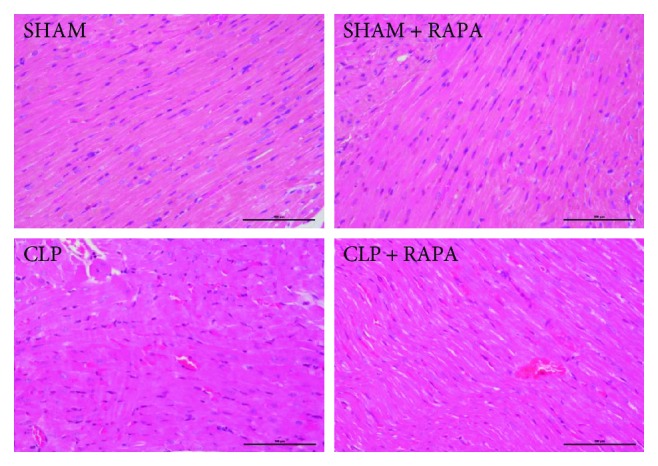
Representative H&E staining of the left ventricle sections. Original magnification, ×200. SHAM, sham operated; CLP, cecal ligation and puncture; RAPA, rapamycin.

H&E staining revealed that the myocardium of SHAM rats had a normal architecture and clear myocyte boundaries, whereas CLP rats after 18 h of sepsis showed marked myocardial injury with myocardial necrosis and interstitial edema adjacent to localized extravasation of red blood cells. These results indicated cardiomyopathy of the sepsis model established by CLP. Pathological damage of myocardial tissue was significantly alleviated by rapamycin treatment (Figure 1).

### 3.2. Rapamycin Alleviates CLP-Induced Cardiac Dysfunction in Rats

**Figure 2:**
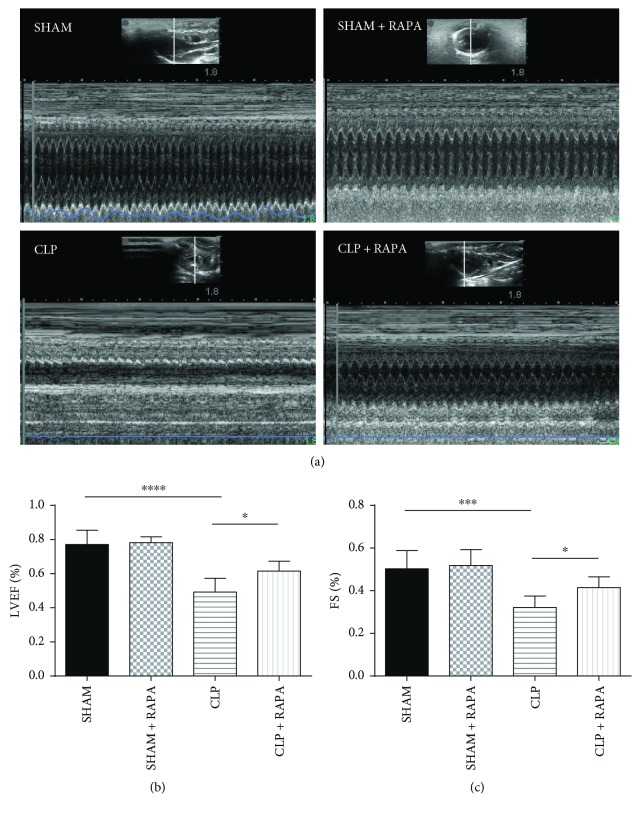
Cardiac functions examined by echocardiography. Representative echocardiographic recordings from the four groups (a). LVEF and FS were measured (b). Mean ± SD, six rats per group, ^∗^*p* < 0.05; ^∗∗∗^*p* < 0.001; ^∗∗∗∗^*p* < 0.0001. LVEF, left ventricle ejection fraction; FS, fraction shortening.

To explore the effects of rapamycin on CLP-induced cardiac dysfunction in rats, the rats were subjected to a noninvasive transthoracic echocardiography. As shown in Figure 2, LVEF and FS were measured at 18 h after treatment. Compared with the SHAM group, cardiac functions as indicated by LVEF (CLP 0.49 ± 0.08 versus SHAM 0.77 ± 0.08, *p* < 0.0001) and FS (CLP 0.32 ± 0.05 versus SHAM 0.50 ± 0.08, *p* < 0.001) were significantly reduced in the CLP group, and rapamycin partially, but significantly, reversed the reduced cardiac dysfunction caused by CLP (LVEF: CLP + RAPA 0.61 ± 0.06 versus CLP 0.49 ± 0.08, *p* < 0.05; FS: CLP + RAPA 0.42 ± 0.05 versus CLP 0.32 ± 0.05, *p* < 0.05).

### 3.3. Rapamycin Reduces Expression of HIF-1a during Septic Cardiomyopathy

**Figure 3:**
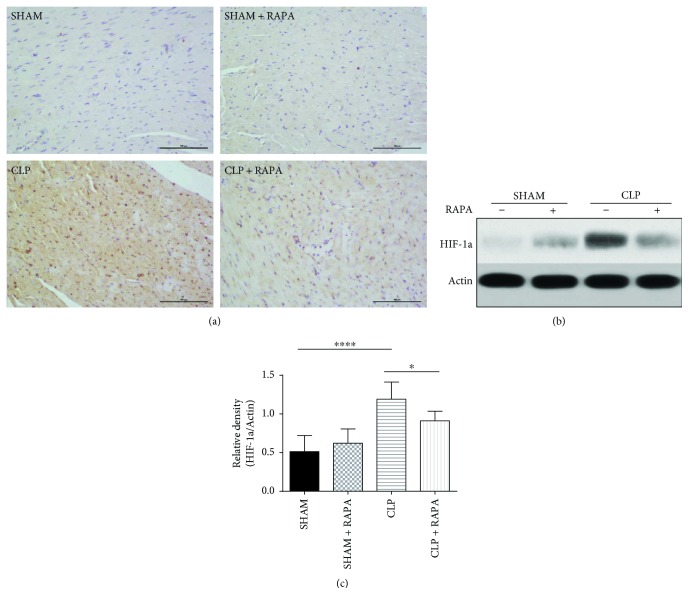
Effects of rapamycin on HIF-1a in the left ventricle 18 h after CLP. (a) The left ventricle sections stained with an anti-HIF-1a antibody. Magnification, ×200. Bar, 100 *μ*m. (b, c) Expression of HIF-1a in the left ventricle. The left ventricle was harvested at 18 h after CLP, and HIF-1a protein levels were quantified by western blotting. Mean ± SD, six rats per group, ^∗^*p* < 0.05; ^∗∗∗∗^*p* < 0.0001.

As shown in Figure 3, HIF-1a expression was significantly increased in CLP rats compared with sham-operated rats (*p* < 0.0001). Interestingly, rapamycin pretreatment of sham-operated rats reduced the expression of HIF-1a compared with that of CLP rats (*p* < 0.05), indicating that rapamycin alleviated myocardial anoxia in septic cardiomyopathy.

### 3.4. mTOR Pathway Activity in the Myocardial Tissue of Model Rats

**Figure 4:**
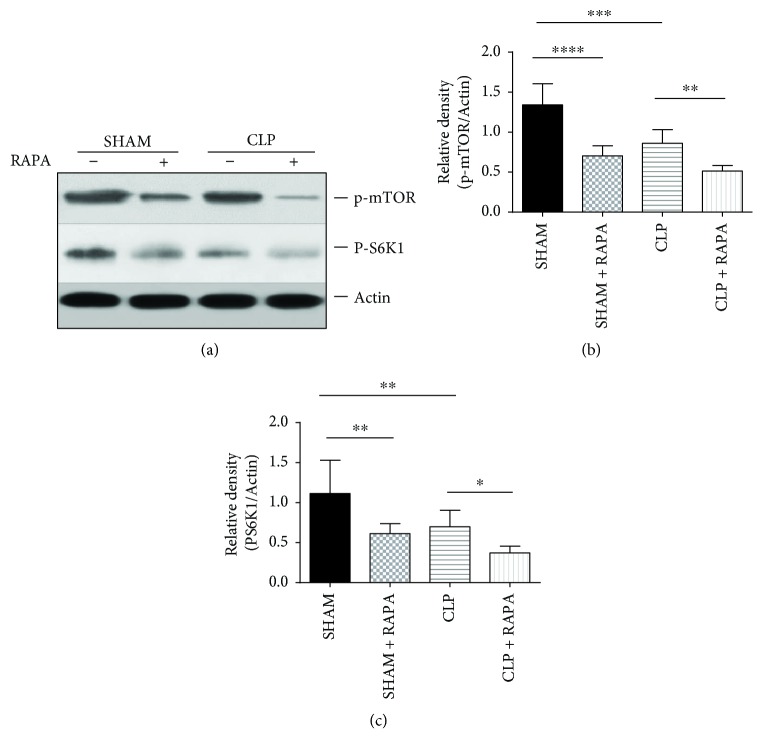
p-mTOR and PS6K1 expression in the left ventricle. The left ventricle was harvested 18 h after CLP, and p-mTOR and PS6K1 protein levels were quantified by western blotting. Mean ± SD, six rats per group, ^∗^*p* < 0.05; ^∗∗^*p* < 0.01; ^∗∗∗^*p* < 0.001; ^∗∗∗∗^*p* < 0.0001.

As expected, the mTOR pathway in the myocardium was significantly inhibited by rapamycin. Interestingly, the activity of the mTOR pathway in the myocardium was dramatically suppressed in CLP rats compared with SHAM rats. The level of p-mTOR (phosphorylation at Ser2448) was decreased in the myocardium of CLP rats (*p* < 0.001) and activation of p70s6 kinase (phosphorylation at Thr389), a direct downstream target of mTOR, was highly suppressed in CLP rats compared with sham-operated rats (*p* < 0.01) (Figure 4). These results indicated that the mTOR pathway might play vital roles in septic myocardial dysfunction.

### 3.5. Relationship between the mTOR Pathway and Autophagy Proteins (LC3-II and p62) in Septic Cardiomyopathy

**Figure 5:**
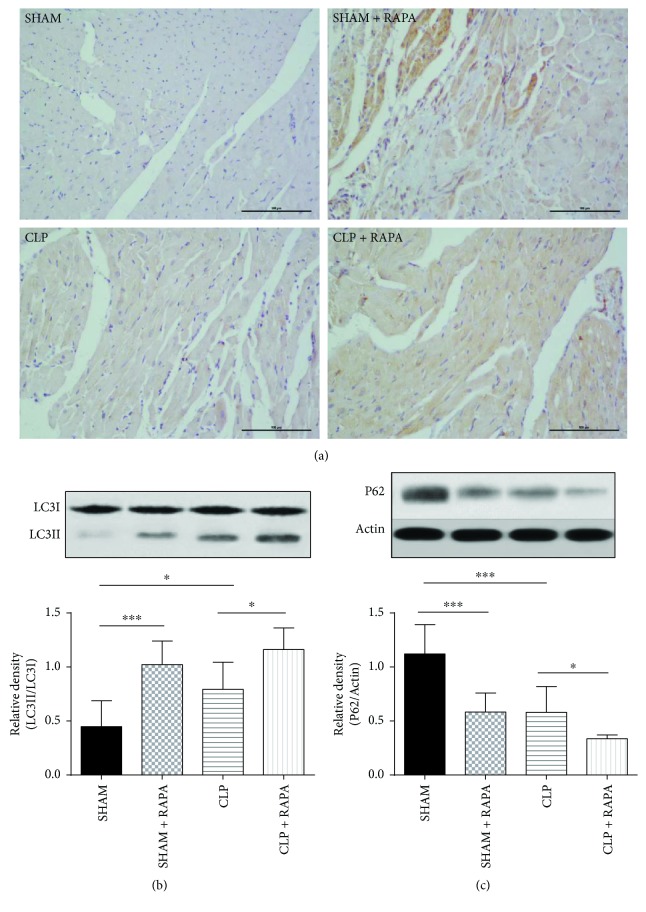
Effects of rapamycin on LC3-II and P62 expression in the left ventricle 18 h after CLP. (a) The left ventricle sections stained with an anti-LC3 antibody. Magnification, ×200. Bar, 100 *μ*m. (b, c) LC3II and p62 expression in the left ventricle. The left ventricle was harvested at 18 h after CLP, and LC3II and P62 protein levels were quantified by western blotting. Mean ± SD, six rats per group, ^∗^*p* < 0.05; ^∗∗∗^*p* < 0.001.

LC3II/LC3I in the left ventricle was significantly increased at 18 h after CLP compared with sham-operated rats (*p* < 0.05). Rapamycin treatment further increased the expression of LC3II in the left ventricle compared with CLP rats without rapamycin treatment (*p* < 0.05) (Figure 5(b)). We also evaluated LC3II in the left ventricle by immunohistochemical staining. As shown in Figure 5(a), the left ventricle sections from CLP rats showed an increase in LC3II expression compared with that from sham-operated rats, and LC3II expression was further increased in CLP + RAPA rats compared with CLP rats. Consistent with the change in LC3II expression of the myocardium, p62 expression in CLP rats was decreased compared with that in sham-operated rats (*p* < 0.001). As shown in Figure 5(c), p62 expression in CLP + RAPA rats was lower than that in CLP rats (*p* < 0.05).

### 3.6. Rapamycin Increases Autophagic Vacuoles in the Myocardium during Septic Cardiomyopathy

**Figure 6:**
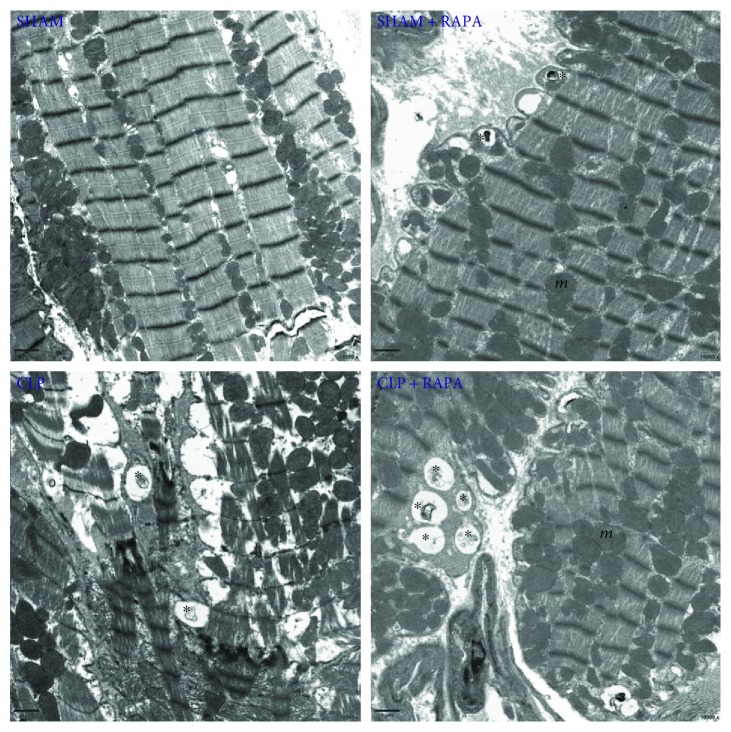
Ultrastructural features of autophagic vacuoles in the left ventricle harvested 18 h after CLP. The myocardium was normal in appearance with a proper mitochondria distribution in sham-operated rats. Myofibrillar disarray was seen in CLP rats. There were more autophagic vacuoles (asterisk) in CLP + RAPA rats compared with CLP rats. Mitochondria (*m*) were seen throughout the cytoplasm. Magnification ×10,000.

As shown in Figure 6, LV tissues from sham-operated rats showed a normal structure, whereas CLP rats had cellular disorganization and myofibrillar disarray. Autophagic processes were observed by transmission electron microscopy. We found more autophagic vacuoles in the myocardium of CLP rats compared with sham-operated rats. We also found that rapamycin further promoted autophagic processes because more autophagic vacuoles were found in CLP + RAPA rats than in CLP rats.

## 4. Discussion

mTOR is a master sensor of energy status and promotes autophagy upon energy depletion [11]. Energy depletion is a major cause of myocardial dysfunction during sepsis [12, 13]. Therefore, the mTOR pathway might play a major role in cardiac dysfunction induced by sepsis. In the present study, we used rapamycin, an inhibitor of the mTOR pathway, to explore the role of the mTOR pathway and the relationship between autophagy and the mTOR pathway in cardiac dysfunction caused by sepsis. We found that the mTOR pathway was inhibited in CLP rats compared with sham-operated rats, and rapamycin significantly alleviated pathological injury and cardiac dysfunction induced by CLP and improved myocardial anoxia in septic cardiomyopathy. We also found that autophagy was activated in CLP rats compared with sham-operated rats, and rapamycin further promoted the autophagic process by affecting the mTOR pathway.

The heart is a high energy-demanding organ because it is required to constantly generate ATP to support the contraction/relaxation cycle. Energy depletion may cause cardiac dysfunction accompanied by changes in nutrient-sensing molecules. For example, in myocardial ischemia, the mTOR signaling pathway is inhibited by AMP-activated protein kinase (AMPK) that is activated by a reduction in cellular ATP levels [18]. Energy depletion also plays a major role in septic cardiac dysfunction induced by mitochondrial dysfunction and reduced cardiac fatty acid oxidation [19]. Our previous studies have found that the mTOR pathway in CD8 (+) T-cell was changed during the sepsis [20]; however, there is little evidence to prove the relevance of mTOR in sepsis-induced cardiac dysfunction [10, 14]. In our study, the mTOR pathway was significantly inhibited at 18 h after CLP. Phosphorylation of both mTOR and pS6K1, downstream targets of mTORC1, was significantly suppressed following CLP challenge. This result is consistent with a study by Li et al. Their study showed that the mTORC1 pathway was inhibited at 12 h after lipopolysaccharide (LPS) (20 mg/kg) injection [14], whereas a recent study indicated that a small dose of LPS (4 mg/kg) overtly suppressed phosphorylation of AMPK and promoted phosphorylation of mTORC1 and S6 in the early stage of sepsis [10]. The reason for the different outcomes may be that in different stages of sepsis, the myocardium has specific energy states which needs to be further explored.

Inhibition of the mTOR signaling pathway has been reported to protect the heart against pathological damage including myocardial infarction and hypertrophy [21–23]. Recently, Li et al. showed that the inhibition of mTORC1 results in cardiac protection against LPS-induced sepsis [14]. However, few studies have supported their results. To explore the role of the mTOR pathway in sepsis-induced cardiac dysfunction, we used rapamycin to inhibit the mTOR signaling pathway. We found that rapamycin significantly alleviated CLP-induced pathological injury and cardiac dysfunction in rats. We also found that CLP rats treated with rapamycin had lower expression of HIF-1a in their myocardium, indicating that rapamycin improved myocardial anoxia in the sepsis model. These results suggest that rapamycin plays a protective role in CLP-induced sepsis cardiomyopathy. Combined with our results showing that CLP inhibited the mTOR signaling pathway in cardiomyocytes, we believe that CLP-induced inhibition of mTOR signaling is beneficial for cardiomyocyte survival, leading to improvement of cardiac functions under sepsis.

We further explored the mechanism by which rapamycin attenuated cardiac dysfunction induced by sepsis. Many studies have shown that the mTOR signaling pathway negatively regulates autophagy. Under nutrient-rich conditions, the mTOR pathway is activated and suppresses autophagy. Conversely, in response to energy depletion, the mTOR pathway is inhibited and autophagy is induced to provide an energy source [11]. As a housekeeping process, autophagy is vital for the normal structure and functions of the heart [7]. Additionally, autophagy plays a critical role in the maintenance of cardiac functions by removing damaged proteins and subcellular organelles under stress conditions [24]. It also plays an important role in the modulation of I/R injury. Evidence suggests that autophagy protects against I/R injury [8]. A recent study has shown that the process of autophagy in cardiomyocytes changes during sepsis, indicating that autophagy might play a major role in septic cardiac dysfunction [9].

In our study, we further explored changes of the autophagy process induced by CLP challenge and the relationship between the mTOR pathway and autophagy. We found that autophagy was activated at 18 h after CLP. The ratio of LC3II/LC3I was increased significantly, indicating that autophagosomal formation was improved at 18 h after CLP. We also found that p62, which inversely correlates with autophagic activity, was decreased significantly, indicating autophagy activation. This finding was confirmed by investigating the ultrastructure of the myocardial cells and finding more autophagic vacuoles in CLP rats. However, in a study by Hsieh et al. [9], they found that the process of autophagy in myocardial cells was not complete at 24 h after CLP. Despite both of our studies revealing elevation of LC3-II, they found that colocalization of LC3 and LAMP1 (a lysosome marker) was decreased at 24 h after CLP. They also observed increased numbers of large autophagosomes containing mitochondria in CLP mice, but few autolysosomes. The reason for the different experimental results may be obtaining myocardial tissues at different stages of sepsis. A recent study demonstrated that autophagy was activated initially in sepsis, followed by a subsequent phase of incompletion in the liver [25], which may be similar in the heart. Even though the time point that we chose was almost the same as that in the study by Hsieh et al., we ligated the cecum at half the distance between the distal pole and base of the cecum to establish the mid-grade sepsis model, whereas they ligated the cecum just below the ileocecal junction for high-grade sepsis. As a result, the myocardial tissue in our study may indicate the condition of autophagy in the early stage of sepsis. We also found that rapamycin further activated autophagy in CLP rats, which was consistent with the improvement of cardiac functions and decreased HIF-1a, indicating that the cardioprotective effect of rapamycin in septic myocardial dysfunction may be mediated by acceleration of autophagy.

In conclusion, inhibition of the mTOR pathway plays a cardioprotective role in septic myocardial dysfunction, and this effect may be mediated by the acceleration of autophagy.

## Data Availability

The details including all figures and raw data used to support the findings of this study are available from the corresponding author upon request.
